# Integrins are required for cardioblast polarisation in *Drosophila*

**DOI:** 10.1186/1471-213X-12-8

**Published:** 2012-02-21

**Authors:** Jessica Vanderploeg, L Lourdes Vazquez Paz, Allison MacMullin, J Roger Jacobs

**Affiliations:** 1 Department of Biology, McMaster University, 1280 Main St. W., Hamilton, ON L8S 4K1, Canada; 2 Life Technologies, Burlington, ON L7L 5Z1, Canada

**Keywords:** Tubulogenesis, Lumen, Apical signaling, Integrin, Slit, Extracellular matrix, Cell polarity, Cell migration, Leading edge

## Abstract

**Background:**

The formation of a tubular organ, such as the heart, requires the communication of positional and polarity signals between migratory cells. Key to this process is the establishment of a new luminal domain on the cell surface, generally from the apical domain of a migratory cell. This domain will also acquire basal properties, as it will produce a luminal extracellular matrix. Integrin receptors are the primary means of cell adhesion and adhesion signaling with the extracellular matrix. Here we characterise the requirement of Integrins in a genetic model of vasculogenesis, the formation of the heart in *Drosophila*.

**Results:**

As with vertebrates, the *Drosophila *heart arises from lateral mesoderm that migrates medially to meet their contralateral partners, to then assemble a midline vessel. During migration, Integrins are among the first proteins restricted to the presumptive luminal domain of cardioblasts. Integrins are required for normal levels of leading edge membrane motility. Apical accumulation of Integrins is enhanced by Robo, and reciprocally, apicalisation of luminal factors like Slit and Robo requires Integrin function. Integrins may provide a template for the formation of a lumen by stabilising lumen factors like Robo. Subsequent to migration, Integrin is required for normal cardioblast alignment and lumen formation. This phenotype is most readily modified by other mutations that affect adhesion, such as Talin and extracellular matrix ligands.

**Conclusion:**

Our findings reveal an instructive role for Integrins in communicating polarising information to cells during migration, and during transition to an epithelial tube structure.

## Background

Vascular endothelia are characterised by a polarised cell architecture, wherein Cadherin based cell junctions establish the integrity of the vessel walls, while the lumen of the vessel is defined by Integrins and an extracellular matrix (ECM) [1,2]. When these vessels first form, grow or are remodeled, the progenitor cells must be less polarised, as they will change neighbours, migrate through other tissues, and respond to local growth cues. During vessel formation, progenitors may have a more mesenchymal organisation, while the differentiated vessel must have stable epithelial polarisation of membrane domains. Vasculogenesis, therefore, can be interpreted in the framework of mesenchymal to epithelial transition [3].

Integrins are transmembrane receptors comprised of pairs of α and β subunits, which link the ECM to the cell cytoskeleton, and mediate cell locomotion, adhesion and signals that affect differentiation and survival [4,5]. There are at least 7 different Integrin dimers expressed in the vascular system, implicated in vascular cell migration, and subsequently in lumen formation [5]. Reduced B1-Integrin function in endothelia, by B1-Integrin antibody in quail embryos [6] or by knockout of B1-Integrin function restricted to mouse endothelia [7] result in a reduced or lost vascular lumen. In other contexts, reduced Integrin function is not sufficient to prevent lumen formation, possibly due to the contributions of other ECM factors [2].

During mesenchymal to epithelial transition, Integrins are thought to regulate migration by activation of intracellular cell polarity signals that modify submembrane protein scaffolds and regulate intracellular protein traffic [8]. The same network of polarising signals, including Rho family GTPases Cdc42 and Rac1, Pak kinase and the Par3-Par6-aPKC complex are also linked to lumen formation in vitro models of lumen formation, such as Madin-Darby Canine Kidney Cells and Human Umbilical Vein Endothelial Cells [9-11]. Therefore, Integrins may play a central role during lumen formation.

*Drosophila *affords multiple models of lumen formation, such as the salivary gland, trachea and dorsal vessel (heart) that enable a genetic dissection of these signal pathways [12-14]. Here we focus on the role of Integrins in the formation of the lumen of the *Drosophila *heart. Despite its relative simplicity, the *Drosophila *heart shares conserved mechanisms of cell fate determination and differentiation with vertebrates, reviewed extensively [15,16]. The *Drosophila *homologues of genes associated with 34 different heart disease genes have been identified, including ones associated with cardiomyopathies, conduction failure, hypertension, atherosclerosis and vascular malformations [17,18].

As with vertebrates, the *Drosophila *heart precursors arise from lateral mesoderm, which migrate medially after specification to meet their contralateral partners, and then assemble a midline vessel [13,19]. The regulators of this collective cell migration event have not been characterised, however genetic and developmental studies illustrate that the precursors (called myocardial cells or cardioblasts, CBs) maintain a close association with the migrating ectodermal cells that provide the inductive signals that establish cardiac fate. The inductive signals are Pyramus, a ligand for the FGF receptor Heartless, and Decapentaplegic, a ligand for the BMP receptor Thickveins [20,21]. Reduced Cadherin function does not affect CB alignment or migration, but the sole Integrin dimer expressed by the CBs (αPS3, βPS), and ligand (Laminin) are required for CB alignment [19,22]. Significantly, the timely migration and alignment of CBs requires the Slit morphogen and Robo receptor, a requirement that interacts genetically with the αPS3 gene *scb*, and the Laminin genes *LanA *and *wing blister *[23-25]. Furthermore, the subsequent formation of a lumen in the *Drosophila *heart requires Slit pathway genes, as well as E-cadherin, Dystroglycan, Syndecan and NetrinB [19,26-28]. These observations suggest that the integrated function of all these cell surface signals are required to complete cell polarisation and establish the luminal ECM. Previous studies suggest that Robo signals establish the luminal domain by excluding Cadherin adhesion [27,29]. Syndecan, recognised as a co-receptor for Slit, is required together with Robo to establish a lumen [28]. However, it is not clear whether Integrins function upstream to apicalise the migrating CBs, or as co-factors with Robo, to help assemble the luminal ECM. Here we characterise changes in CB polarisation and heart morphogenesis subsequent to genetically altered Integrin function. We identify a role for the αPS3, βPS1 Integrin dimer in both the early establishment of the apical, pre-luminal domain, as well as a requirement upon Robo for maintained apicalisation of proteins required for lumen formation. This suggests that Robo dependent lumen formation acts downstream of other cell polarising signals.

## Results

**Figure 1:**
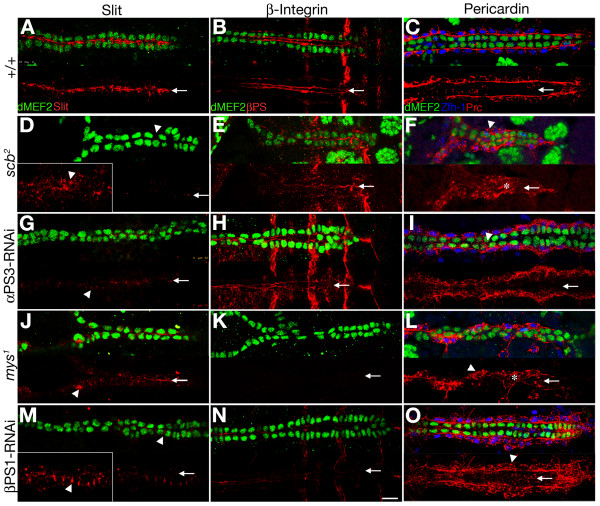
**αPS3 Integrin is required for apicalisation of cardioblasts**. Slit is located in the heart lumen (arrow) in stage 17 wildtype embryos (A). The level of immuno-detected Slit (red) is much lower in *scb^2 ^*mutants (D), and *mys^1 ^*mutants (J) or if α or β Integrin levels are reduced by dsRNA knockdown (scbRNAi in G and mysRNAi in M, directed by dMEF2-GAL4). Residual Slit is not apicalised, and is found on lateral cell surfaces (arrowheads in insets of D and M). Most of the immunolabel for βPS1 Integrin is concentrated on the luminal surface in wildtype (red, B). Levels of βPS1 are reduced in *scb *mutants (E) and subsequent to scbRNAi expression (H), however, some luminal label remains. No βPS1 is detected in *mys^1 ^*mutants (K), and trace levels of βPS1 remain after mysRNAi expression (N). Pericardin, synthesized by the pericardial cells (labeled with Zfh-1 antibody, blue in C, F, I, L, O) is restricted to the basal surface of the CBs in wildtype (red, C). Reduced Integrin function in *scb *and *mys *mutants (F, L), or subsequent to scbRNAi (I) or mysRNAi (O) expression results in Pericardin re-distribution around the entire pericardial cell perimeter (arrowheads, F, L, O), as well as between some CBs (arrowhead in I), and less at the basal surface of the CBs. Midline Pericardin marks medially displaced pericardial cells (asterisk in F, L). The CB nuclei here and subsequent fluorescence images are identified with antibody to dMEF2 (green), except where CB nuclei are labeled with β-galactosidase for the B2-3-20 enhancer trap in G, H, I, M, N, O (green). Anterior is to the left in all panels, and the arrow marks the dorsal midline. All images were generated at the same gain. The gain in the insets is adjusted to visualise low intensity immuno-label signals. Calibration: 10 microns

The *Drosophila *heart vessel is formed by the dorsal and medial migration of lateral somatic mesodermal cardioblasts (CBs). The migration of this mesoderm is linked to dorsal closure, which is the tandem dorsal migration of lateral ectoderm to envelop the gut, which also replaces a transient embryonic tissue, the amnioserosa on the dorsal surface of the embryo [30]. Previous studies have established a role for the αPS3/βPS1 Integrin dimer in the displacement and involution of the amnioserosa, as well as the assembly of the heart vessel [22]. Involution of the amnioserosa, and dorsal closure is incomplete in embryos lacking zygotic function of either αPS3 (*scb*) or βPS1 (*mys*), complicating an assessment of heart morphogenesis. We have assessed the mutant heart in two manners: in zygotic mutants, and after dsRNA mediated reduction of Integrin levels. Zygotic mutants for the amorph *scb^2 ^*had complete dorsal closure of abdominal segments 6 to 8, which contains most of the heart chamber, so our analysis focused on these segments. In wildtype embryos, and embryos heterozygous for *scb^2^*, two rows of CB were aligned across the midline, and the morphogen, Slit was restricted to the heart lumen (Figure 1A). Embryos mutant for αPS3 (*scb^2^*), in contrast, revealed less regular alignment of CB nuclei, with displaced CBs forming blisters across or beside the midline being a common feature (Figure 1D). Levels of Slit were reduced, and were not concentrated apically. Similarly, zygotic mutants for βPS1 (*mys^1^*) had blisters and gaps in the CBs, and reduced Slit accumulation (Figure 1J, Table 1), although apicalisation was better preserved.

**Table 1:** Leading Edge polarisation deficits in the heart region of the dorsal vessel

*Genotype*	*yw*	*mys^1^*	*scb^2^*	*mys RNAi*	*scb RNAi*	*n (per genotype)*
Embryos with CB displacement	4	**16**	**12**	10	**12**	*20 embryos*
Apical/Non-Apical Slit	8.6 ± 1.2	***2.6 ± 0.3***	***1.8 ± 0.1***	***2.2 ± 0.2***	***2.1 ± 0.1***	*9 embryos*
Leading Edge Activity, %	41 ± 3	***12 ± 2***	***16 ± 3***	42 ± 2	36 ± 3	*12 LEs*

Slit apicalisation is expressed as a ratio of the average immunofluorescence intensity in the apical region, divided by the intensity of non- apical heart fluorescence. Means shown ± standard error. Data sets significantly different from *yw *or *yw, UAS-moe-mCherry/+, tup-GFP, dMEF-GAL4/+ *controls are in bold italic if *p *< .001 and in bold if *p *< .05 by Mann-Whitney *U *test

We found that βPS1 Integrin was concentrated in the luminal domain of wildtype stage 17 hearts (Figure 1B, arrow) with lower levels on the basal surface of the CBs. In the absence of α Integrin, β Integrin is not properly targeted to the cell surface [31]. Accordingly, in *scb *mutants, levels of βPS1 were reduced, with relatively more immunolabel outside the luminal domain (Figure 1E). βPS1 antigen was not detected on CBs of zygotic *mys^1 ^*mutants (Figure 1K).

Pericardin is a collagen-like protein secreted by the pericardial cells, which concentrates at the CB-pericardial cell interface [32]. In wildtype, Pericardin labeling marked a continuous structure at the base of the CBs (Figure 1C). In *scb *mutants, Pericardin label more frequently surrounded the pericardial cell (arrowhead, Figure 1F), and was distributed discontinuously on the CB surface. Embryos mutant for *mys *have Pericardin distributed irregularly over the pericardial cell surface, and pericardial cells were displaced towards the midline (asterisk, Figure 1L).

As an independent approach to assessing Integrin function in the CB, we have reduced αPS3 and βPS1 protein levels in the mesoderm, with targeted expression of dsRNA (in *dMEF2-GAL4/UAS- scbRNAi *embryos in Figure 1G-I, and in *dMEF2-GAL4/UAS- mysRNAi *embryos in Figure 1M-O). We obtained qualitatively similar results with 2 *scbRNAi *strains and 2 *mysRNAi *strains (see Methods). Dorsal closure was normal after Integrin knockdown in the mesoderm, and CBs from all segments met their contralateral partners. However, small gaps or clumps in alignment are observed (Figure 1G, M). As in the mutant, the level of Slit was reduced (Table 1), and strikingly, Slit was detected between ipsilateral cells, a membrane domain from which it is normally excluded (arrowheads, Figure 1G, M) [19,29]. We further assessed the distribution of βPS1 Integrin (Figure 1H, N) and Pericardin (Figure 1I, O) and encountered phenotypes intermediate between wildtype and *scb^2^*. Lower levels of βPS1 were observed subsequent to *scb RNAi *expression, possibly because of some perdurant αPS3 protein. Very low levels of βPS1 labeling subsequent to mysRNAi (Figure 1N) suggests that quantities of maternally contributed βPS1 are limited. Nevertheless, for both Integrin knockdown genotypes, Slit and Pericardin distribution was abnormal (Figure 1G, I, M, O).

Considered together with the data from the zygotic mutants, we conclude that Integrin function is required for orderly alignment of the CBs, is essential for establishing an apical or luminal domain for CBs, and polarises Pericardin on the pericardial cells. The reduction in the level and apical deposition of Slit in mutants indicates that Integrins are required to target or stabilise this morphogen apically.

### Integrins are required for heart lumen formation

**Figure 2:**
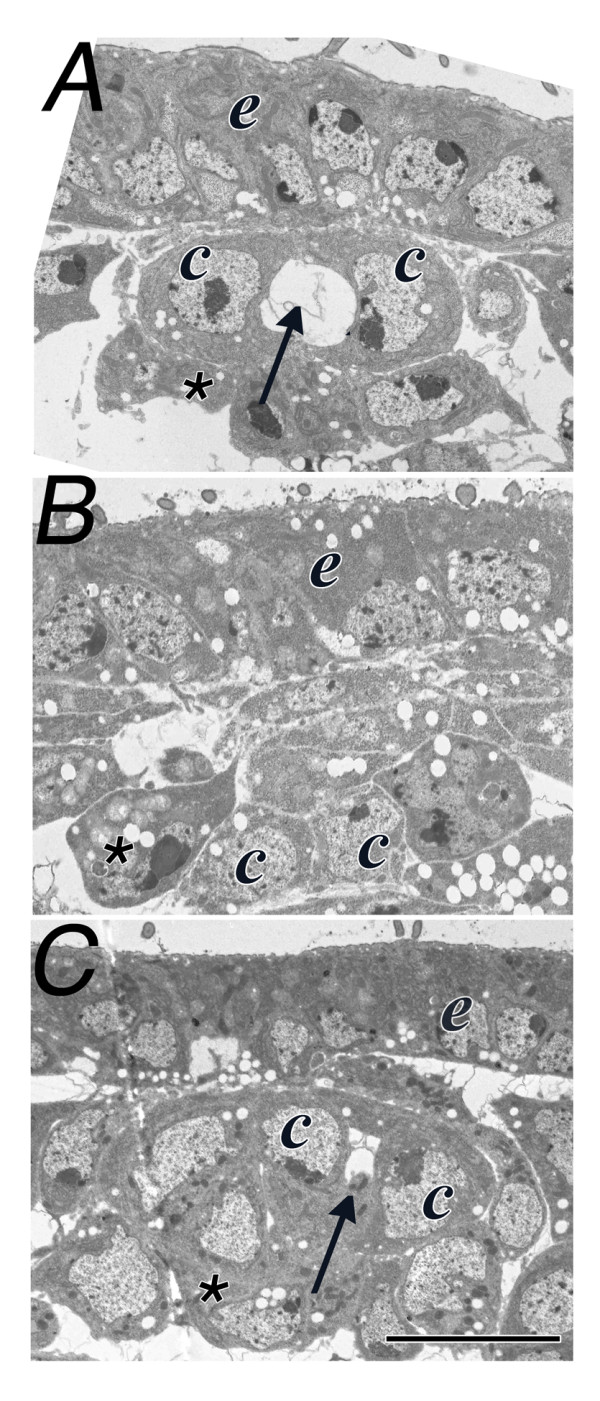
**αPS3 Integrin is required for lumen formation**. The lumen of the *Drosophila *heart is developed in stage 17 wildtype embryos (arrow), between contralaterally apposed cardioblasts (c). No lumen develops between cardioblasts in *scb^2 ^*mutant embryos (B), however, there is partial restoration of the lumen (arrow, C) in *scb^2 ^*mutant embryos expressing an αPS3 transgene (*scb^2^/scb^2^; dMEF-GAL4/UAS-αPS3*). Sections were sampled between abdominal segments 6-8. Ectodermal cells (e) and amnioserosa cells (asterisk) are identified; calibration: 5 microns

**Figure 3:**
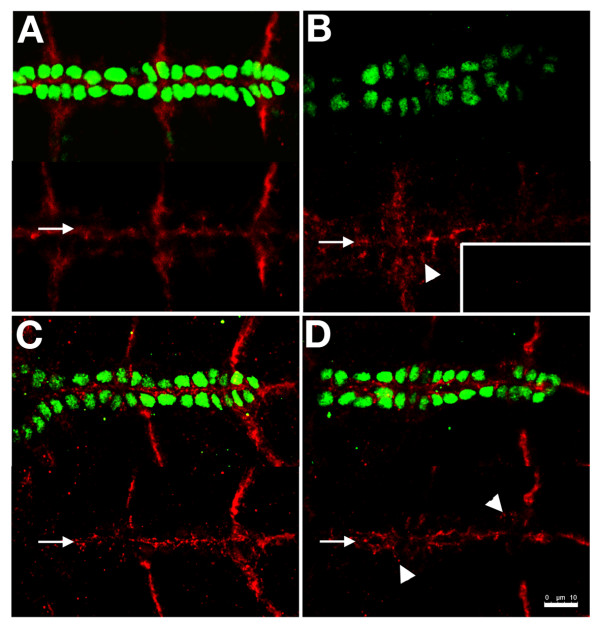
**Expression of an αPS3 transgene in the CBs restores apicalisation of Robo**. Like the ligand Slit, its receptor Robo is apicalised in wildtype (red, A). In *scb^2 ^*homozygotes, Robo levels are dramatically reduced (B, unadjusted gain shown in inset), however at increased gain, Robo at the midline (arrow) and between ipsilateral cells (arrowhead) is detected. In *scb/scb*; *dMEF-GAL4/UAS-scb *embryos, expression of αPS3 only in somatic mesoderm is sufficient to restore apical restriction of Robo (C). Rescue of *scb^2^/scb^2 ^*with a cytoplasmically truncated αPS3 transgene yields partial restoration of midline Robo (arrow, D), although Robo between ipsilateral cells is detected (arrowheads). Calibration: 10 microns

Previous studies have established that apicalisation of Slit is required to establish a lumen in the *Drosophila *heart [23,25]. Given the mislocalisation and reduced levels of Slit subsequent to reduced Integrin function, we sought to determine if lumen formation was similarly affected. A well developed lumen was visualised in cross-sections of stage 17 hearts, and no lumen was detected between contralaterally apposed CBs in the heart region of *scb^2 ^*mutants (Figure 2A, B). However, expression of a αPS3 transgene in the CB of *scb^2 ^*mutants partially restored lumen formation (Figure 2C). These data are consistent with a model wherein Integrin acts to apicalise Slit binding and signaling, which is a pre-requisite for lumen formation. We addressed this model directly by determining whether restoring αPS3 expression to the CBs in a *scb *mutant would restore apicalisation of the Slit receptor, Robo. In contrast to wildtype (Figure 3A), Robo levels are reduced and incompletely apicalised in *scb *mutants (Figure 3B and inset). As expected, mesodermal expression of αPS3 in *scb *mutants did not restore normal dorsal closure. However, Robo, was concentrated apically (Figure 3C), and no longer laterally as seen in *scb^2 ^*mutants. Truncation of the short cytoplasmic domain of αPS2 locks the Integrin into an "excessively active" high affinity receptor, forming ectopic adhesions [33]. Rescue of *scb *mutants with a similarly truncated transgene was sufficient to restore apicalisation of Robo, also expanding the apical area of Robo expression (Figure 3D).

### Integrins are required for CB polarisation

**Figure 4:**
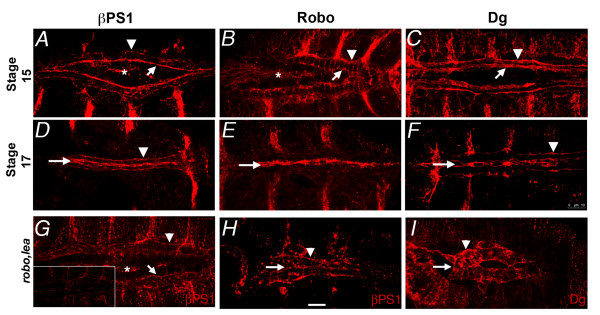
**Integrins apicalise early during CB polarisation**. During CB migration, Integrins (βPS1 immunolabel) are predominantly located on the advancing apical edge (A, arrow), with low levels on the basal trailing edge (arrowhead). Integrin is not detected on lateral CB surfaces, yet at the same stage, Robo is detected on apical, lateral and basal membrane domains (B). Dystroglycan is excluded from lateral cell surfaces, and is concentrated at both apical and basal membranes (C). At stage 17, migration is complete, and the heart lumen is formed. The apical and basal distribution of Integrin has not changed (D), however, Robo (E) and Dystroglycan (E) are now almost entirely apical. Apicalisation of Integrin is reduced in *robo, lea *mutants at stage 15 (G) and 17 (H). The inset in (G) shows the intensity of immuno-labeling at the same gain as the other βPS1 panels. Dystroglycan apicalisation is similarly affected in *robo, lea *mutants (I). The asterisks in stage 15 panels identifies βPS1 label in the amnioserosa. Arrows mark apical surfaces, and arrowheads mark basal surfaces of the CBs. Calibration: 10 microns

During their medial migration, the CBs are in an intermediate state of polarisation. The CBs adhere to their ipsilateral neighbours, which is likely Cadherin based [19], and the advancing and trailing edges interact with the ECM, which is likely Integrin based. However, when the CBs meet their contralateral partners, the leading edge of the cells will develop a luminal domain that concentrates Slit and Robo. Given that a luminal domain fails to develop in *scb *mutants, and that luminal markers require *scb *function to apicalise, we speculated that Integrins may be instructive in establishing the location of the luminal domain. We therefore sought to determine how the distribution of Integrins reflected the transition from a migratory phenotype to epithelial differentiation. In stage 15 embryos, before the leading processes of the CBs make contact across the midline, the βPS1 Integrin was localised primarily on the leading edge of the migrating CBs (Figure 4A, arrow) while much less label was detected on the trailing edge (Figure 4A, arrowhead). The preponderantly apical distribution was not significantly altered once the lumen was formed (Figure 4D). In contrast, Robo protein was located on apical, lateral and basal surfaces of the CB during migration (Figure 4B), and is entirely luminal by stage 17 (Figure 4E). Dystroglycan, another apical membrane protein required for lumen formation, was also excluded from lateral membrane domains at both stages, although more basal labeling was evident during migration that was reduced after lumen formation (Figure 4C, F, arrowheads).

**Figure 5:**
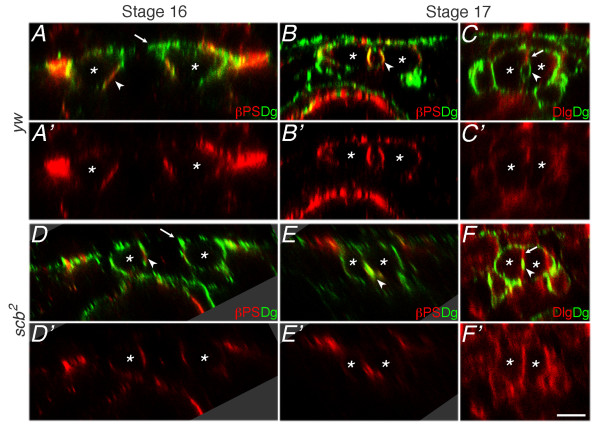
**Integrins concentrate at the presumptive luminal domain**. Transverse optical sections from abdominal segments 6-8 of immunolabeled embryos reveal that βPS1 Integrin (red) is concentrated ventral to the leading edge of migrating CBs (arrowhead, A, A'), where the lumen forms at stage 17 (arrowhead, B, B'). Dystroglycan (Dg, green) outlines most of the cell surface during migration, and concentrates at the lumen surface later than Integrin (A, B). However, Dystroglycan is excluded from the cell junction between contralateral CBs, which instead accumulates Discs-Large (Dlg, arrow, red) at stage 17 (C, C'). Zygotic *scb^2 ^*mutants accumulate lower levels of βPS1 Integrin apically during migration (arrowhead, D, D'). Integrin and Dystroglycan are both located apically at stage 17, but lumen formation fails (arrowhead, E, E'). An adherent domain marked by continuous Dlg labeling (arrow, F, F') indicates that no lumen forms, although a pocket of apical Dystroglycan labeling is still seen (arrowhead, F). The dorsal leading process apparent in *yw *migrating CBs (arrow, A) is missing in *scb^2 ^*mutants (arrow, D). Dorsal at top; asterisks label approximate location of CB nucleus. Scale bar is 5 microns

Previous studies demonstrate that the apical surface of mature CBs are subdivided into a junctional (J) domain and a luminal (L) domain [27]. We sought to determine whether formation of these domains required Integrin function, and whether early Integrin apicalisation reflected early establishment of the luminal domain. We therefore examined migrating CBs in cross-sectional view to determine whether Integrin was spatially restricted to a subdomain of the leading edge membrane before midline fusion. The dorsal limit of a wildtype CB at stage 16 extended Dystroglycan labeled membrane towards the midline (Figure 5A, arrowhead), yet this extension was missing in *scb *mutants (Figure 5D, arrowhead). βPS1 Integrin accumulated immediately ventral to the dorsal extension during migration, and this domain contributed to the Integrin rich luminal membrane at stage 17 (Figure 5A, B, arrowheads). The accumulation of Integrin was reduced in *scb *mutants, but a presumptive luminal domain was still apparent (Figure 5D, E, arrowheads). At stage 17, Dystroglycan labeling outlined the heart lumen (L domain; Figure 5B, C, arrowhead), and Discs-large accumulated at the adhesive junction between contralateral CBs (J domain; Figure 5C, arrow). When Integrin function was reduced, the Discs-large domain is uninterrupted, and a small domain expressing both Discs-large and Dystroglycan was observed (Figure 5F).

These data suggest that early Integrin aggregation presages the formation of the luminal domain during CB migration, and Integrin may therefore be instructive in localizing factors like Robo, also required to establish the lumen. These data do not exclude the possibility that other apical signals work in concert with Integrin. We also explored the possibility that Robo signaling augments apical signaling. In embryos lacking function of both Robo1 and Robo2 (*robo^1^lea^54-14^*), βPS1 and Dystroglycan distribution were remarkably less polarised at both stages 15 and 17 (Figure 4G, H, I), even though Robo was not normally apically concentrated until stage 17 (Figure 4E). At stage 15 it is unlikely that CBs can detect contralateral Slit. Therefore, autocrine Slit signaling may contribute to CB polarisation during migration.

### Integrins are required for robust leading edge motility

**Figure 6:**
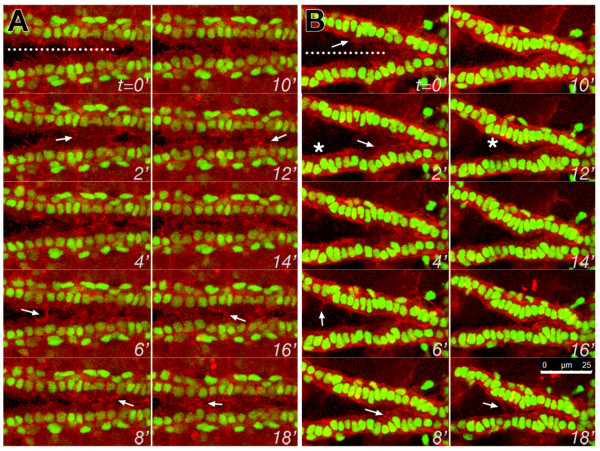
**Leading Edge membrane activity requires Integrin function**. The dynamic activity of the leading edge of migrating CBs was monitored in living embryos, labeling membrane actin with *dMef-GAL4 *regulated *UAS-moesin-mCherry*, while marking the CBs with *tup-F4-GFP*. In early stage 16, wildtype CBs of segments 6-8 are 20 microns apart, and extend highly dynamic lamelipodial and filopodial processes to contact their contralateral partners (arrows in A). Although zygotic mutants of *mys^1^* generate similar cell processes (arrows in B), most of the leading edge is quiescent (asterisks), and medial migration is slower. Images are taken at 2 minute intervals; the dorsal midline at t = 0 is marked with a dotted line. Scale: 25 microns

Cell migration requires polarised membrane and cytoskeletal trafficking, and instructive polarising signals may require outside-in signals from Integrins [8,34]. Of the apical markers examined, only Integrin is concentrated at the apical leading edge before stage 16. Embryos mutant for *scb^2 ^*appear to lack a leading membrane process (Figure 5D). We therefore sought to determine whether the exploratory activity of the leading edge requires normal Integrin function. Leading edge activity is effectively assessed with fluorescently tagged moesin, which binds to submembrane actin, outlining lamellipodia and filopodia [35]. We compared the density and length of membrane processes of wildtype CBs (Figure 6A) with those of zygotic *mys *(Figure 6B) and *scb *mutants (Table 1). Although leading edge activity was clearly present in *mys*, only 12% of the leading edge was active, while in contrast, membrane activity was more vigorous in wildtype, incorporating 41% of the leading edge of CBs (Table 1). A phenocopy of this effect subsequent to dsRNA interference of *mys *or *scb *could not be generated, possibly due to the presence of an additional UAS target (UAS-mCherry). The low level of leading edge activity in Integrin mutants may reflect maternally contributed Integrin function, or compensation by other apicalising signals. It remains to be resolved whether Integrin signaling from the pre-luminal domain affects leading edge activity, or whether Integrins function in the leading processes, but are not detectable after histochemical processing.

### Integrins interact genetically with genes for adhesion signaling

**Figure 7:**
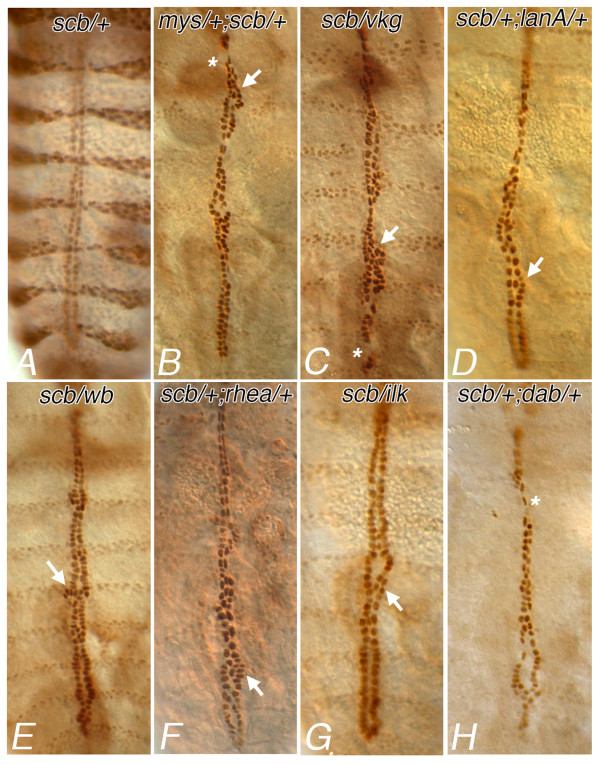
**αPS3 Integrin interacts with mutations in genes for adhesion and adhesion signaling**. Cardioblast position at stage 17 is visualised for embryos zygotically and maternally heterozygous for both *scb^2 ^*and zygotically heterozygous for an interacting gene. Embryos haplosufficient for *scb *have normal heart assembly (**A**). If additionally heterozygous for the gene for βPS1 Integrin (*mys^1^*), the continuity (asterisks) and alignment (arrows) of the CBs is disrupted (**B**). A similar phenotype is seen in embryos also heterozygous for collagen IV (*vkg^[p1003-8]^*, **C**), whereas mutation in two Laminin chains (*LanA *^9-32^, **D **and *Lamininα2*, or *wb^SF11^*, **E**) affect CB alignment, but without effect on continuity. Genetic interactions are revealed with a haplosufficiency in adhesion second messengers associated with Integrin, such as Talin (*rhea^1^*, **F**) and ILK (**G**), as well with second messengers associated with guidance signaling, such as Disabled (*dab^M54-R1^*, **H**). CBs labeled with the B2-3-20 enhancer trap. Anterior at top

A contribution by Integrin function to CB migration or polarisation can be revealed through genetic interactions between *scb *and mutations in genes that act in the same, or a converging pathway. We have surveyed possible interactions with genes coding components of the ECM, and with genes that act to mediate adhesive or morphogenetic signals. Similar phenotypes were observed in embryos heterozygous for *scb^2^*, and also heterozygous for mutations in βPS1 or known Integrin ligands Collagen IV (*vkg*) Laminin chains α3,5 and α1,2 (*lanA, wb*) and Tiggrin (*tig*, not shown) (Figure 7B, C, D, E respectively). Phenotypic interactions were characterised by interruptions in the continuity of each CB leading edge, evidenced by either small gaps (Figure 7, asterisks) or spans or clumps of CBs, three or more cells across (Figure 7 arrows). We also screened for interactions between *scb *and genes for intracellular factors that mediate cytoskeletal responses to signals from the membrane. Interestingly, embryos doubly heterozygous for *scb *and Talin (*rhea*) had a phenotype similar to that seen for the ECM gene interactions, suggesting that Talin, which links Integrins to the actin cytoskeleton, mediates the effects of adhesion to the ECM (Figure 7F). In contrast, perturbations in heart morphology were less stereotyped for genes believed to affect actin remodeling, and acting downstream of Robo (*dab, dock*, and *abl*; Figure 7H, and data not shown) or Integrin (*ilk*, Figure 7G). These data suggest that Integrin function in CB alignment is more sensitive to factors affecting adhesion than to changes in cytoskeletal signaling.

## Discussion

Morphogenesis of the *Drosophila *heart provides an accessible genetic model to dissect the signals that orient migrating mesenchymal cells, and enable the cells to transform to a differentiated, stable epithelial structure with luminal and basal identity. A diversity of genes has been identified that are required for lumen formation in the heart. They include genes encoding ECM proteins, such as Laminin A, homophilic adhesion, such as Cadherin, and genes associated with mediating cell guidance, such as Slit or NetrinB [19,26-28]. This work establishes that Integrins are also required for CB polarisation- during cell migration, for apical leading edge motility, and during lumen formation. A lumen fails to develop in the hearts of embryos lacking *scb *function, but the luminal domain can be restored by expression of αPS3 in the CBs of a *scb *mutant.

Although Robo is believed to be key to the establishment of the luminal domain, the mechanisms that localise Robo function are unclear [27,29]. Our previous studies establish a close functional relationship between Robo function and Integrins, in both axon guidance, and in heart morphogenesis [23,36]. Apical accumulation of βPS1 Integrin precedes apicalisation of the proposed lumen determinants, Slit and its receptor, Robo. Furthermore, in *scb^2 ^*mutants, Robo and Slit do not accumulate apically, and in fact, are found on lateral cell surfaces, associated with Cadherin based adhesion. Restoring *scb *function with either normal or high affinity αPS3 restores Robo apicalisation- suggesting that regulating Integrin affinity for the ECM is not critical for its apical signal.

Robo signaling prevents local accumulation of Cadherin in both neurons and CBs - and in the heart, it has been proposed that this is the basis of generating an non-adherent luminal domain [29,37]. Our data suggests that Robo signaling must act in concert with Integrin to restrict Cadherin from the apical domain. In the salivary gland model of lumen development, Cadherin is removed from the luminal domain by endocytosis, employing Rho family GTPases and Pak1 [38], which in turn, are downstream of Integrin and Robo signals [39,40].

Given that mutation of any one of 7 cell surface receptors (Cadherin, Integrin, Robo, Neurexin, Syndecan, Dystroglycan and Unc5) is sufficient to block lumen formation, it is likely that cooperative signaling defines the luminal domain and luminal differentiation [19,24,26-28]. Of the 7 required receptors, Robo, Syndecan, Dystroglycan, Integrin and Unc5 locate to the luminal domain. Integrin, its ligand Laminin [41], and Unc5 [26] concentrate in the presumptive luminal domain before the others, and likely are instructive. Our data also suggests that the presumptive luminal domain is defined by Integrin accumulation during migration, and before contact with contralateral CBs.

At stages 15 and 16, βPS1 Integrin localises apically when Robo is still expressed on all membrane domains. Nevertheless, at this stage, polarised Integrin distribution requires Robo function. It is possible that Integrins suppress Robo turnover at the apical surface only, and Robo signaling stabilises the Integrins, culminating in the apical aggregation of Robo seen by late stage 16. Leading edge membrane motility becomes most pronounced during stage 16. Loss of Integrin function reduces the fraction of the Leading Edge that is active. In contrast, loss of *robo *function reduces Leading Edge activity uniformly, and filopodia are rare ([27,29] and unpublished observations), suggesting that Integrin dependent accumulation of Robo augments membrane activity.

The sensitivity of *scb *function to ECM genes Collagen IV and Laminin suggest that Integrin effects on apicalisation are adhesion dependent. The genetic interaction of *scb *with *rhea *(Talin) further suggests that assembly of a complex of ligand-bound Integrin, linked to the actin cytoskeleton through Talin, both stabilises the apical membrane domain, and enables Robo accumulation. An understanding of how the luminal domain differentiates requires future study on signaling by this multi-receptor complex.

## Conclusions

We have characterised the contribution of Integrin function to cell migration and lumen formation in a mesodermal organ, the *Drosophila *heart. Integrins are required to maintain the alignment of the migrating CB leading edge cells, and are also required for normal levels of leading edge motile membrane activity. A pre-luminal domain, first revealed by early Integrin accumulation, is defined before the CBs meet their contralateral partners. Integrin accumulation is required to stabilise the morphogen Slit, and its receptor Robo, to the pre-luminal domain. This is consistent with Robo signaling contributing to leading edge membrane activity. Even through Robo does not aggregate apically in migratory CBs, its expression is required to stabilise Integrins in the pre-luminal domain.

We confirm an earlier report that αPS3 Integrin is required for heart lumen formation [22]. Integrin function is also required to localise a uniform ECM around the heart, as visualised with Pericardin. Genetically, this function is most sensitive to genes required for adhesion, such as for Talin, and ECM ligands. Slit signaling during axon guidance and salivary gland migration is also sensitive to Integrin function [14,36]. Integrins may have a conserved function in establishing membrane domains rich in Robo to enable localised morphogen signaling.

## Methods

### Drosophila melanogaster Strains

The mutant strains *scb^2^, scb^01288^, mys^1^, mew^M6^, lea^2^, robo^1^lea^54-14^, sli^2^, wb^SF11^, wb^09437^, dock^04723^, vkg^177-2^, ilk^ZCL3111^, ilk^2^, dab^M54-R ^*and *rhea^1 ^*were obtained from the Bloomington Stock Center. The laminin allele *lan^A9-32 ^*was provided by C. Goodman (Berkeley), *ras^5703 ^*by D. Montell (Johns Hopkins University), *lea^5418 ^*by C. Klämbt (Münster), *vkg ^p1003-83 ^*by N. McGinnis (University of Massachusetts) and *tig^X ^*was provided by T. Bunch (University of Arizona). Multiple alleles of each gene were tested when possible. All stocks were maintained over β-galactosidase marked balancers. The following GAL4 and UAS lines were used for ectopic expression and knockdown experiments: *dMef-GAL4, P[TRiP HMS00043](mys)*, and *P[TRiPJF02696](scb) *from the Bloomington Stock Center, *UAS-scb RNAi *(KK106326) and *UAS-mys RNAi *(KK100518) from the Vienna Stock Center. *UAS-scab *was constructed using 5'-TGG CGT AGA ATT CAT CTG TTG-3' (EcoRI site) and 5'-TCA CGA TCT AGA GGA CAT TC-3' (XbaI site) as primers for a PCR reaction to amplify the full length protein ScabA from the original EST clone (RE41844, Berkeley *Drosophila *Genome Project). The lacZ enhancer trap line B2-3-20 used to visualize the cardial cell nuclei was provided by E. Bier [42]. *tup-F4-GFP *was provided by R. Schulz [43] and *UAS-moe-mCherry *was provided by T. Millard [35].

### Antibodies, immunohistochemistry and microscopy

Embryonic development proceeded at 25°C for all experiments, except the RNAi experiments, performed at 29°C. Immunohistochemistry techniques were adapted from [44]. Embryos were collected, dechorionated, fixed and incubated in primary antibody diluted in phosphate buffered saline (PBS) containing 0.1% Triton X and 10% normal goat serum. β-galactosidase was detected with a chicken antibody (1:150) [23]. The following monoclonal antibodies were obtained from the Developmental Studies Hybridoma Bank, under the auspices of the NICHD: anti-Robo (1:30), anti-Slit (1:30), anti-Discs Large (1:30), anti-Pericardin (1:30), anti-βPS1 (1:30). Dystroglycan antiserum (1:600) was kindly provided by W. Deng [45]. To visualize muscle cell nuclei, polyclonal anti-MEF2 antibodies were generated by injecting a His tagged MEF2 fusion protein (amino acids 1-168, construct provided by H. Nuygen) [46] into New Zealand white rabbits by use of standard conditions. Embryos were incubated in fluorescent secondary (1:150 dilution, Alexa 488, 594, 647 and 546; Molecular Probes). β-Gal was detected using biotinylated secondary antibody (1:150; Vector Laboratories) followed by incubation with Vector Laboratories Elite ABC and 3,3-Diaminobenzidine Tetra hydrochloride (DAB, Gibco-BRL). Embryos were visualized by confocal microscopy using a Leica SP5 microscope. All images shown are projections of 3 to 5 optical sections, processed using OpenLab, ImageJ and Adobe Photoshop.

Timelapse microscopy was performed from a *tup-F4-GFP, dMEF-GAL4, UAS-moe-mCherry *background to visualise cardioblast nuclei and the cardioblast leading edge, employing the hanging drop protocol [47], on a Leica SP5 microscope.

### Electron microscopy

Dechorionated embryos were fixed in heptane equilibrated with 25% glutaraldehyde (Fluka) in 0.1 M Sodium Cacodylate. Embryos were manually devitellinated in 4% paraformaldehyde and 2.5% glutaraldehyde in cacodylate buffer, post-fixed in 1% osmium tetroxide, and stained in uranyl acetate before embedding in Epon-Araldite [48]. Lead stained 0.1 μm sections were examined on a JEOL 1200EXII microscope. We examined over 120 sections from specimens of each genotype.

## Abbreviations

aPKC: atypical Protein Kinase C; BMP: Bone Morphogenic Protein; CB: Cardioblast; ECM: Extra Cellular Matrix; FGF: Fibroblast Growth Factor; MEF2: Myocyte Enhancing Factor 2.

## Competing interests

The authors declare that they have no competing interests.

## Authors' contributions

JV and LVP carried out all the genetic and phenotypic studies. AM generated the αPS3 transgenes and transgenics, and some of the data in Figure 7. JRJ generated the data in Figure 2 and drafted the manuscript. All authors read and approved the final manuscript.
